# Intraoperative patellar tracking assessment during image-based robotic-assisted total knee arthroplasty: technical note and reliability study

**DOI:** 10.1051/sicotj/2024037

**Published:** 2024-10-29

**Authors:** Cécile Batailler, Salomé Greiner, Hanna-Lisa Rekik, Flora Olivier, Elvire Servien, Sébastien Lustig

**Affiliations:** 1 Orthopaedics Surgery and Sports Medicine Department, FIFA Medical Center of Excellence, Croix-Rousse Hospital, Lyon University Hospital 69004 Lyon France; 2 Univ Lyon, Claude Bernard Lyon 1 University, IFSTTAR, LBMC UMR_T9406 69622 Lyon France; 3 Stryker France SAS 69330 Pusignan France; 4 LIBM – EA 7424, Interuniversity Laboratory of Human Movement Science, Université Lyon 1 69003 Lyon France

**Keywords:** Total knee arthroplasty, Patellar tracking, Image-based robotic-assisted system, Anterior compartment, Personalized alignment

## Abstract

*Introduction*: Restoration of the anterior knee compartment is increasingly studied with the development of personalized surgery. However, evaluating the patellar tracking during the surgery is still subjective and at the surgeon’s discretion. This study aimed 1) to describe the assessment of the patellar tracking during robotic-assisted total knee arthroplasty (TKA), 2) to describe a new measurement technique for evaluating the evolution of this patellar tracking, and 3) to assess its reliability and repeatability. *Method*: This monocentric study assessed the evolution of patellar tracking for 20 robotic-assisted TKA. The sharp probe was used to perform patellar tracking in all the arcs of knee flexion before and after the bone cuts. The patella positioning was recorded every 10° of flexion between the full extension and 90° knee flexion and was assessed in the coronal and sagittal planes. For the measurements of the patellar tracking, we used a sagittal view and a coronal view of the knee on the MAKO software. From these two views, the difference between the patellar tracking before and after the bone cuts with the definitive implants was measured. Two independent reviewers performed the measurements to assess their reliability. To determine intraobserver variability, the first observer performed the measurements twice. *Results*: The mean age was 68.7 years old ± 5.2 [61; 75], the mean body mass index was 28.8 kg/m^2^ ± 4.2 [21.4; 36.2], the mean HKA angle was 176.3° ± 3.7° [174.1.4; 179.7]. The radiographic measurements showed very good to excellent intra-observer and inter-observer agreements (0.60 to 1.0). *Conclusion*: This new measurement technique assessed the evolution of patellar tracking after TKA with good inter and intra-observer reliability.

## Introduction

In total knee arthroplasty (TKA), both surgeon and patient expectations are continuously rising. The contemporary objectives of primary TKA include not only improved functional outcomes but also the capability to engage in more demanding daily activities, such as kneeling and recreational sports, which were previously unattainable [1]. As a common cause of discomfort, pain, or preclusion from these activities, almost 50% of these patients point to the anterior part of the knee [2, 3].

Restoration of the anterior knee compartment is being studied increasingly with the development of personalized surgery. Even minor imperfections in the restoration of this compartment can lead to patellar maltracking, severe pain, and poor functional outcomes [4]. During knee flexion, the patella’s movement is primarily guided by the retinaculum between 0 and 30° and subsequently by the shape of the trochlear groove during mid-flexion and high flexion [5, 6]. This dynamic relationship is influenced by various factors, including surgical techniques (particularly implant positioning, femoral, and patellar sizing) and implant characteristics (such as trochlear depth and shape, sagittal curvature, and patellar component design) [7, 8]. New robotic-assisted technologies now enable surgeons to address the patellofemoral and tibiofemoral compartments independently and to reconsider thinking about the anterior knee compartment. Indeed, thanks to the preoperative CT scan, the shape of the trochlea is materialized on the screen during the planning. The femoral implant can be superimposed and positioned precisely in the same position as the native trochlea if wanted. Consequently, recreating the physiologic position and depth of the trochlea groove appears easier with this robotic assistance.

However, the assessment of patellar tracking during surgery is still subjective and at the surgeon’s discretion. Image-based robotic-assisted systems can now visualize patellar tracking intraoperatively; however, no standardized measurement of this tracking has been established.

This study aimed 1) to describe the assessment of the patellar tracking during robotic-assisted total knee arthroplasty (TKA), 2) to explain a new measurement technique for evaluating the evolution of this patellar tracking, and 3) to assess its repeatability and reliability.

The hypothesis was that this measurement technique was reliable and consistently repeatable.

## Material and methods

### Study design

This retrospective study included 20 primary total knee arthroplasties performed using an image-guided robotic system in a varus population. Prior to surgery, each patient underwent a dedicated CT scan with 3D reconstructions.

### Surgical technique

All surgeries were performed by an orthopaedic surgeon with more than five years of experience in robotic-assisted TKA and more than 200 cases per year.

The MAKO robotic platform planning software (MAKO, Stryker Corporation, Mahwah, NJ, USA) allowed preoperative implant planning using a patient-specific CT-based bone model and virtual implant templates. As shown previously, the 3D implant model followed the bone anatomy and was accurate to within 1 mm [9].

- *First assessment of patellar tracking (before bone cuts)*

**Figure 1:**
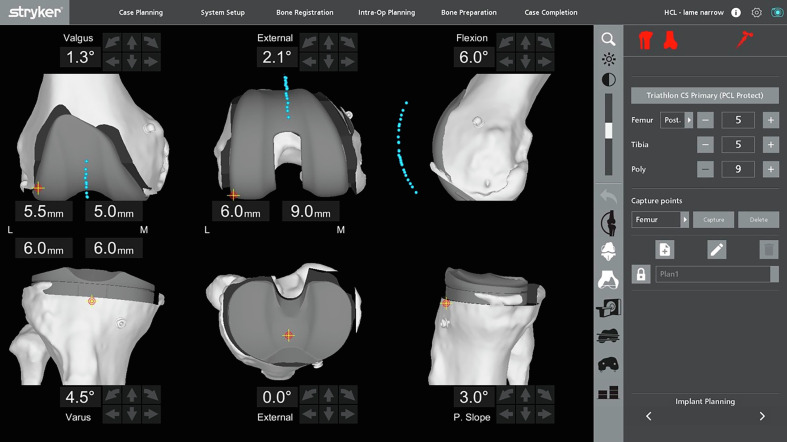
Patellar tracking before the bone resection on the CT scan of the knee planning.

The patellar tracking is assessed before the bone resections. The patella is reduced in front of the femur. It’s essential to make a landmark on the anterior face of the patella to use the same landmark at each patellar tracking assessment (Video 1). This landmark, performed with the electric scalpel, is located in the middle of the patella in the mediolateral and proximal-distal axis. The sharp probe is used to perform the patellar tracking because it is more stable on the bone (Video 1). It is crucial not to constrain the patella with the probe to ensure the real patellar positioning in all the arcs of knee flexion. The knee goes from full extension and is flexed progressively (Video 2). The MAKO engineer records the patella positioning every 10° of flexion between the full extension and 90° knee flexion (Video 2, Figure 1).

The patella’s positioning can be assessed in the coronal (mediolateral) and sagittal (anteroposterior) positions.

- *TKA planning and bone cuts*

The positioning of the implant followed the principles of functional alignment described previously [10]. If a lateral translation of the patella is identified before the planning during the patellar tracking, the femoral implant can be positioned a little more lateral and with higher lateral rotation. Bone cuts were then executed with the robotic arm.

- *New assessment of patellar tracking (after bone cuts)*

The final step is the verification of the functional plan. Limb alignment and gap sizes can be assessed clinically and with real-time feedback from the robot, either with trial or definitive implants, or both. The patellar tracking is again evaluated to analyze the evolution of the patellar tracking.

In the case of patellar resurfacing, this assessment is performed before (after the bone resections) and after the patellar resurfacing. The evaluation before the patellar resurfacing allows us to adjust the patellar button positioning if needed.

It is essential to use the same patellar landmark with the sharp probe. The knee goes again from full extension and is flexed progressively without constraint on the patella. The MAKO engineer records the patella positioning every 10° of flexion between the full extension and 90° knee flexion, as before the bone cuts (Video 3).

**Figure 2:**
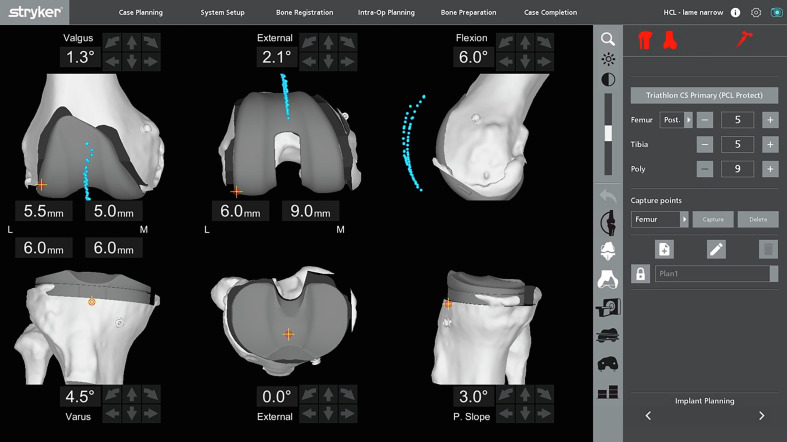
The new assessment of the patellar tracking after the bone resections and with the definitive implants. The two patellar tracking (before and after the TKA) can be compared on the same screening.

The patella’s positioning can be compared to the preoperative positioning in the coronal (mediolateral positioning) and sagittal planes (anteroposterior positioning) (Figure 2).

### Measurement technique

All measurements were made using the MAKO robotic platform planning software (MAKO, Stryker Corporation, Mahwah, NJ, USA). A calibrated millimetre scale allowed accurate and reliable measurements with an accuracy of 1 mm.

**Figure 3:**
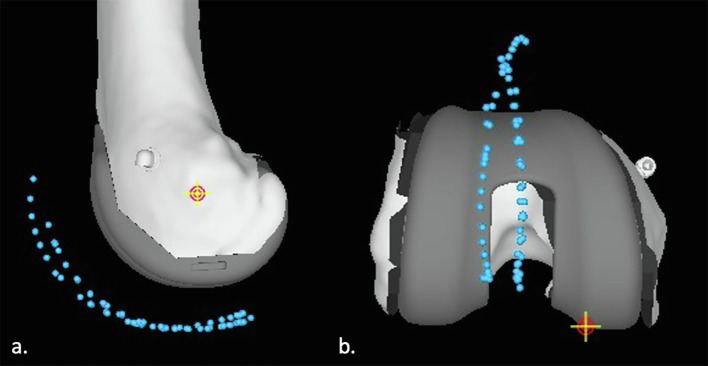
(a) Sagittal and (b) coronal views of the knee with patellar tracking before and after the bone cut.

**Figure 4:**
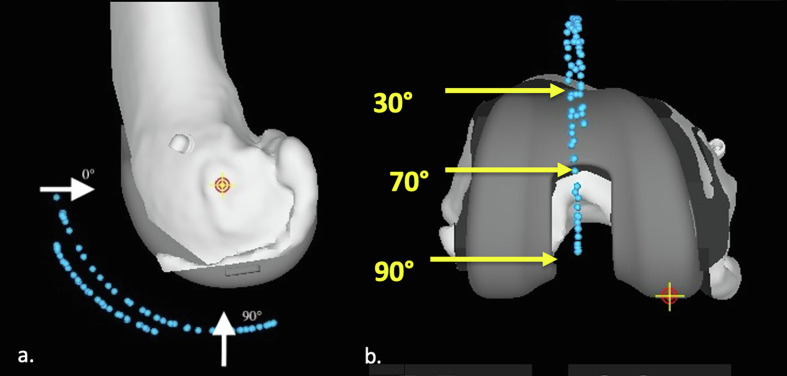
(a) Sagittal view of the knee with the localization of the measurements at 0° and 90°. (b) Coronal view of the knee with the measurements localized at 30°, 70°, and 90°.

For the measurements of the patellar tracking, we used a sagittal view (Figure 3a) and a coronal view (Figure 3b) of the knee on the MAKO software. On these two views, we measured the difference between the patellar tracking before the bone cuts and after the bone cuts with the definitive implants. On the sagittal view, we measured this anteroposterior difference every 10° between 0° and 90°, considering 0° as the vertical position and 90° as the horizontal position distally compared to the knee rotation centre (Figure 4a). On the coronal view, we measured this mediolateral difference in three positions corresponding to 30°, 70° and 90° (Figure 4b).

A difference inferior to 1 mm was considered null. Measurements were performed by two independent reviewers (an orthopaedic surgeon and a medical student) for all measurements to assess the reliability of each measurement. To determine intra-observer variability, the second observer measured the patients twice, four weeks apart. Both observers were trained on the MAKO platform to learn the measurement technique.

### Statistical analysis

Statistical analysis was performed using the XL STAT software (Version 2021.2.1, Addinsoft Inc., Paris, France). An intraclass correlation coefficient evaluated the inter- and intra-observer reliabilities of the measurements. The strength of agreement for the kappa coefficient was interpreted as follows: <0.20 = unacceptable, 0.20–0.39 = questionable, 0.40–0.59 = good, 0.60–0.79 = very good, and 0.80–1 = excellent [11]

## Results

The mean age was 68.7 years old ± 5.2 [61; 75], the mean body mass index was 28.8 kg/m^2^ ± 4.2 [21.4; 36.2], the mean HKA angle was 176.3° ± 3.7° [174.1.4; 179.7].

The values of the evolution of patellar tracking are summarized in Table 1. There was a tendency to obtain a patella closer to the femur at the proximal part of the femoral implant. All the mediolateral modifications were a decrease in the patellar lateralization.

**Table 1:** Values of the evolution of patellar tracking.

	Mean	Standard deviation	Range
Sagittal 0°	4.2	1.6	2.4–6.3
Sagittal 10°	4.1	1.8	2.2–6.4
Sagittal 20°	4.2	2.0	1.8–6.7
Sagittal 30°	3.3	1.5	1.6–5
Sagittal 40°	3.2	1.5	1.5–5
Sagittal 50°	2.1	1.9	0–4.4
Sagittal 60°	1.2	1.6	0–4.1
Sagittal 70°	0	0	0
Sagittal 80°	0	0	0
Sagittal 90°	0	0	0
Coronal 30°	1.6	1.9	0–3.8
Coronal 70°	1.5	2.6	0–5.9
Coronal 90°	1.3	2.2	0–5

The radiographic measurements showed very good to excellent intra-observer and inter-observer agreements (Table 2).

**Table 2:** Intraobserver and inter-observer coefficients for the evolution of patellar tracking.

	Intra observer ICC	Agreement	Inter observer ICC	Agreement
Sagittal 0°	0.83	Excellent	0.81	Excellent
Sagittal 10°	0.62	Very good	0.63	Very good
Sagittal 20°	0.61	Very good	0.60	Very good
Sagittal 30°	0.76	Very good	0.70	Very good
Sagittal 40°	0.72	Very good	0.69	Very good
Sagittal 50°	0.71	Very good	0.73	Very good
Sagittal 60°	0.82	Excellent	0.80	Excellent
Sagittal 70°	1	Excellent	1	Excellent
Sagittal 80°	1	Excellent	1	Excellent
Sagittal 90°	1	Excellent	1	Excellent
Coronal 30°	0.74	Very good	0.75	Very good
Coronal 70°	0.83	Excellent	0.79	Very good
Coronal 90°	0.84	Excellent	0.81	Excellent

*Note*: The strength of agreement for the kappa coefficient was interpreted as follows: < 0.20 = unacceptable, 0.20–0.39 = questionable, 0.40–0.59 = good, 0.60–0.79 = very good, and 0.80–1 = excellent.

## Discussion

The main finding of this study was the description of a new technique for intraoperative measurement of patellar tracking evolution during total knee arthroplasty. This technique has demonstrated both reliability and repeatability.

Using classic mechanical instrumentation, the surgeons usually focus on the tibiofemoral joint, overlooking the patellofemoral joint’s complexity. Surgeons typically manage this by adjusting the rotation and lateralization of the femoral component. This unawareness of the anterior compartment can lead to early complications or revisions due to patellofemoral problems [12]. The advantage of this new patellar tracking assessment technique is its intraoperative application, allowing for adjustments before the implantation of definitive components. Based on real-time assessment, surgeons can modify femoral and tibial implant positioning (e.g., lateral position, rotation) and the placement of the patellar button. In addition, this could provide new insight for decision-making regarding patella resurfacing or not and how patella cut should be performed (direction in 3D and amount of bone to be removed). Every modification can be assessed immediately, including its impact on patellar tracking. This could decrease the complications of the extensor mechanism after TKA, particularly the patellar instability.

Personalized alignment techniques, performed with an image-based robotic-assisted system, have demonstrated superior understanding and restoration of the trochlear groove compared to mechanical or kinematic alignment methods [10, 13]. Mechanical alignment often results in the trochlear groove being positioned farthest from its native anatomy, while kinematic alignment can lead to unsafe coronal implant positioning in approximately 13% of cases and internal rotation of the femoral component beyond 3° in over 25% of cases [10]. By contrast, the functional alignment had only 3.2% of patients outside coronal and 1.7% outside rotational safe zones [10]. Nevertheless, these personalized surgeries tend to understaff the trochlea and the anterior compartment, as reported in an in-vitro study using an image-based robotic system [10]. Recent studies showed the same tendency with a patellar tracking closer to the femoral implant between 0° and 50° [14, 15]. This study also showed significant patellar tracking modification in the sagittal plane during early flexion between 0° and 40° (3.2–4.2), likely due to proximal trochlear understuffing of the femoral implant. In the coronal plane, we also observed a reduction of the lateral translation for some patients with a preoperative lateral subluxation of the patella. Interestingly that this lateral translation can be assessed during surgery and corrected immediately.

Shatrov et al. described a similar analysis with an image-less robotic-assisted system [16]. They described the patella centre of rotation, with a high variability of modification after TKA in a small cohort. Further analyses are necessary to better understand the evolution of patellar tracking according to surgical planning.

This measurement technique has demonstrated its inter and intra-observer reliability and repeatability. The precision of measurement, facilitated by preoperative CT scans, was accurate to within 1 mm. The main difficulty in the measurement was the difference inferior to 1 mm because it cannot be assessed. However, a difference inferior to 1 mm can be considered insignificant clinically. Prior training with the robotic software was essential for observers’ accurate interpretation of measurements.

This study had several limitations. First, the MAKO system was necessary to measure the CT scan with the implants in place. Secondly, the measurements are based on CT scans and do not account for cartilage thickness. However, cartilage thickness did not impact the measurements since we evaluated differences between the two periods. The first measurements were difficult to perform, underscoring the importance of thorough training for observers using the MAKO software. This study did not evaluate the impact of these measurements on clinical outcomes; further studies are needed to explore these implications.

To our knowledge, this is the first study to describe a measurement technique for evaluating the evolution of patellar tracking after image-based robotic-assisted total knee arthroplasty. This study did not aim to interpret the evolution of patellar tracking but to develop a reliable measurement technique.

## Conclusions

This new measurement technique evaluated the evolution of patellar tracking after total knee arthroplasty with good intra and inter-observer reliability. A further study should be conducted to correlate the development of patellar tracking after TKA with the functional results.

## Data Availability

Data associated with this article cannot be disclosed due to legal reasons.

## Supplementary materials

**Video 1**: Video showing the landmark for the patellar tracking in the middle of the patella and the sharp probe on the patella to track the patellar positioning during knee flexion. **Video 2**: Recording of the patellar positioning in extension and flexion before the bone cuts. **Video 3**: Recording of the patellar positioning in extension and flexion after the bone cuts with the trial implants. Supplementary material is available at https://www.sicot-j.org/10.1051/sicotj/2024037/olm.
